# Criteria and models for the distribution of casualties in trauma-related mass casualty incidents: a systematic literature review protocol

**DOI:** 10.1186/s13643-017-0538-z

**Published:** 2017-07-12

**Authors:** Ali Ardalan, Mohammad Reza Khajehaminian, Sayed Mohsen Hosseini Boroujeni, Amir Nejati, Abbasali Keshtkar, Abbas Rahimi Foroushani, Omid Mahdi Ebadati E.

**Affiliations:** 1 Department of Disaster Public Health, School of Public Health, Tehran University of Medical Sciences, Tehran, Iran; 2 Department of Health Sciences Education Development, School of Public Health, Tehran University of Medical Sciences, Tehran, Iran; 3 Department of Biostatistics and epidemiology, School of Public Health, Tehran University of Medical Sciences, Tehran, Iran; 4 Department of Mathematics and Computer Sciences, Kharazmi University, Tehran, Iran

**Keywords:** Mass casualty incident, Casualty distribution, Casualty evacuation, Pre-hospital Emergency Care

## Abstract

**Background:**

One of the most critical practices in mass casualty incident management is vacating the victims from scene of the incident and transporting them to proper healthcare facilities. Decision on distribution of casualties needs to be taken on pre-developed policies and structured decision support mechanisms. While many studies tried to present models for the distribution of casualties, no systematic review has yet been conducted to evaluate the existing models on casualty distribution following mass casualty incidents. A systematic review is therefore needed to examine the existing models of patient distribution and to provide a summary of the models. This systematic review protocol is aimed to examine the existing models and extracting rules and principles of mass casualty distribution.

**Methods:**

This study will comprehensively investigate existing papers with search phrases and terms including “mass casualty incident”, distribution, evacuation, and Mesh terms directly corresponding to search phrases. No limitations on the type of studies, date of publication, or language of the relevant documents will be imposed. PubMed, Web of Science, Scopus, and Google Scholar will be searched to access the relevant documents. Included papers will be critically appraised by two independent reviewers. The data including incidents type, scene characteristics, patient features, pre-hospital resources, and hospital resources will be categorized. Subgroup analysis will be conducted when possible.

**Discussion:**

To the best of our knowledge, no study has yet addressed the effects and interaction of contributing factors on the decision-making processes for casualty’s distribution. This is the first study that comprehensively assesses and critically appraises the current models of casualty distribution. This study will provide evidences about models and criteria for casualty distribution following mass casualty incidents.

**Systematic review registration:**

PROSPERO Registration Number: CRD42016049115

**Electronic supplementary material:**

The online version of this article (doi:10.1186/s13643-017-0538-z) contains supplementary material, which is available to authorized users.

## Background

The World Health Organization (WHO) defines a mass casualty incident as “an event which generates more patients at one time than locally available resources can manage using routine procedures” [1]. The chaotic nature of mass casualty incidents can generally put extreme stress on healthcare systems [2]. The dynamism and transformation characteristic of these incidents necessitate making pre-hospital management decisions under severe time constraints [2, 3]. Because the number of severely damaged patients in these incidents is significantly higher and the nature and severity of the injuries are more complex, challenges faced by healthcare systems during a mass casualty incident are quite different from routine practices [2].

The numerous definitions available for a mass casualty incident indicate an environment of limited resources that further challenges the management of such incidents [4]. In mass casualty incident, any imbalance between the needs and the quickly available resources can overcrowd admission and treatment centers, such as hospital emergency departments, operating rooms, and ICUs [5]. Studies show that long waiting periods in emergency departments and high bed occupancy rates are often inevitable in these situations. Overcrowded hospitals not only affect the emergency department but also put extreme pressure on other critical departments of the hospital [6].

Evacuating the wounded from the scene of a mass casualty incident and transferring them to proper healthcare centers is a highly critical task [2, 7]. To avoid putting extreme pressure on any single hospital due to the transfer of large numbers of wounded and prolonging the waiting period in critical hospital departments, the decision about how to distribute the patients among the available healthcare centers should be made with great care [8]. The capacity of hospitals for caring for the severely damaged is associated with the flow of admitted patients. As a result of sudden increase in the number of patients admitted to a single hospital or the surge of patients, the quality of patient care might be jeopardized [9].

The decision about the distribution of patients among different hospitals is normally taken by the incident commander based on the availability of resources, details of the incident, and the triage data [10]. The commander and others involved in evacuating the wounded from the scene of the incident should be capable of making a rapid decision about distributing the patients [2].

Research into real-time decision-making processes in dynamic situations suggests that most of these decisions are made based on previous experiences. With a greater experience of managing mass casualty incidents comes better decision-making in stressful conditions. Nonetheless, mass casualty incidents are a rare occurrence, and few emergency managers may face these incidents at a large scale during their career [3]. Managing large numbers of patients in mass casualty incidents can therefore be considered a serious challenge for disaster managers, as they need to analyze several factors simultaneously [6]. This fact makes most disaster response managers unable to perform this duty [4].

Studies on the distribution of patients following mass casualty incidents suggest the need for policy-based decision instead of relying on the emergency managers’ experience and skills [2, 4, 6]. Given the discussed points and the consequences of the poor management of patient distribution and in order to avoid the geographic effect and overload a single hospital, a systematic method of patient distribution is required to assign the casualties to the different hospitals available [4].

Compared to the other daily experiences of disaster managers and hospital staff, mass casualty incidents are relatively rare occurrences. Models provide a proper means of describing the effects of selecting different approaches and available resources. Models might help managers to identify deficiencies in the management of mass casualty incidents, which most of these deficiencies are not clear prior to the actual incident and during routine performances [8]. Although many studies have been conducted to present models for the distribution of patients, no systematic reviews have yet been conducted to evaluate the existing models. A systematic review is therefore needed to examine the existing models of patient distribution and to provide a summary of the models.

## Methods

### Aims/objectives

#### Primary objectives

The primary objectives of this systematic review include:

1. Investigating the criteria for casualty distribution in trauma-related mass casualty incidents
2. Reviewing the existing models of casualty distribution in trauma-related mass casualty incidents

#### Secondary objectives

The secondary objectives of this study include:

1. Determining the criteria for patient distribution in trauma-related mass casualty incidents based on scene characteristics, patient features, pre-hospital, and hospital resources.
2. Assessing the models and criteria for patient distribution in trauma-related mass casualty incidents by type of incident (air, railway, RTIs, etc.)
3. Assessing the models and criteria for patient distribution in trauma-related mass casualty incidents by method of model assessment (real-world, simulation, exercise, etc.)

### Study registration

This systematic review will be conducted using the preferred reporting items for systematic review and meta-analysis protocols (PRISMA-P) 2015 (See Additional file 1) [11]. This protocol was developed using the PRISMA Protocol Checklist and was registered with the PROSPERO, international prospective register of systematic reviews with the registration number: CRD42016049115.

### Eligibility criteria

#### Type of studies

This study investigates all the available criteria and models for distributing and transferring casualties of trauma-related incidents from the scene of incident to healthcare centers irrespective of the level of trauma. This study will impose no restrictions on the type of studies conducted on the subject and no limitations on the date of publication or language of the relevant documents as well.

Relevant studies might include actual environments, simulated environments, and exercises. Studies on evacuating patients from the hospital, the inter-hospital transfer of patients, the air evacuation of the wounded from the scene of accident, the evacuation of injured from the battlefield, and the evacuation of the wounded from scenes off the ground, such as marine scenes, will not be included.

#### Type of participants

The study participants include all the casualties of trauma-related mass casualty incidents. The study imposes no age restrictions. Moreover, the casualties of chemical, biological, radiological, and nuclear incidents are excluded from the study.

### Information sources and the search strategy

Databases such as PubMed, Web of Science, Scopus, and Google Scholar will be searched to access the relevant documents. No limitations are imposed on the publication status of the extracted documents. MeSH terms will first be used to extract all the entry terms associated with the phrase ‘mass casualty’ and also in combination with terms such as ‘evacuation’ and ‘distribution’. The generated combination of keywords will be searched in the All Field bar with no limitations on search environment. Databases will be searched using following syntaxes to obtain relevant papers:

#### PubMed

All Field ((((Casualty Incident AND Mass) OR (Casualty Incidents AND Mass) OR (Incident AND Mass Casualty) OR (Incidents AND Mass Casualty) OR (Mass Casualty Incident) OR (Mass Casualties) OR (Casualties AND Mass) OR (Casualty AND Mass) OR (Mass Casualty)))) AND ((distribut* OR evacuat*))

#### Scopus

(ALL(Casualty Incident AND Mass) OR ALL(Casualty Incidents AND Mass) OR ALL(Incident AND Mass Casualty) OR ALL(Incidents AND Mass Casualty) OR ALL(Mass Casualty Incident) OR ALL(Mass Casualties) OR ALL(Casualties AND Mass) OR ALL(Casualty AND Mass) OR ALL(Mass Casualty)) AND (ALL(distribut*) OR ALL(evacuat*))

#### Web of Science™

(TS = (Casualty Incident AND Mass) OR TS = (Casualty Incidents AND Mass) OR TS = (Incident AND Mass Casualty) OR TS = (Incidents AND Mass Casualty) OR TS = (Mass Casualty Incident) OR TS = (Mass Casualties) OR TS = (Casualties AND Mass) OR TS = (Casualty AND Mass) OR TS = (Mass Casualty)) AND (TS = (distribut*) OR TS = (evacuat*))

### Study records

#### Selection process

The reviewers will briefly investigate the documents to identify relevant papers based on title and abstract. The results screened by title and abstract are entered into the EndNote and duplicates will be removed. Following determining the relevant papers, two well-informed reviewers about the study background will use the study’s eligibility criteria to examine the full-texts of any potentially pertinent studies identified so far. Any disagreement between the two reviewers is resolved through a group discussion and consensus. A third reviewer is asked for assistance in the case of an unresolved disagreement. To find other potentially relevant documents, the references of the extracted articles will also be examined. The corresponding authors of the articles included in the study will also be asked to introduce any studies which they know that are completed or still in progress and meet the eligibility criteria of the present research. Using the remarks made by specialists of this field, key journals of the field will also be identified and manually reviewed to find relevant papers published over a 10-year period. In addition to the above-mentioned items, textbooks introducing rules and principles will be reviewed to identify relevant documents.

#### Data collection process

After completing the process of searching literature, data extractors will be given access to the full text of the studies. Each data extractor will extract the original data independently based on a pre-developed form. The study variables to be extracted include the country which the study is originated, the department conducted the study, the type and method of the study, the principles and rules of patient distribution regarding to scene characteristics, patient features, pre-hospital resources, and hospital resources.

### Risk of bias in the individual studies

Given the lack of restrictions on the type of initial studies selected, no specific tools for quality assessment can be introduced in this step. To evaluate the methodological quality of the initial studies, however, quality assessment tools introduced in STROBE will be used depending on the type of the study. In this step, every study is presented along with its proper assessment tool to the two independent reviewers. Any disagreement between the reviewers about the quality of the study will be resolved by consensus; otherwise, a third reviewer will be asked for advice on the quality of the article.

### Dealing with missing data

The corresponding author of included papers will be contacted by email and requested to get data, if failing to extract any of the intended data from the full text of the articles. The study will be excluded from the research if the missing data cannot be retrieved.

### Data analysis

The present study explores the existing criteria and models for the distribution of casualties and extracts model components. If possible, a subgroup analysis is conducted using the existing models and based on the casualty distribution method. If the outcomes of the model application have been reported, a comparison will be made. The components extracted from all the models will be classified in terms of incidents type, scene characteristics, patient features, pre-hospital resources, and hospital resources.

## Ethics

The study has ethical approval from Institute Review Board of Tehran University of Medical Sciences with the registration Number IR.TUMS.SPH.REC.1395.509.

## Discussion

Studies have shown that the management of patient distribution among healthcare centers is not a simple but rather challenging practice [7, 12], as the nearest hospitals are often overloaded by receiving patients in numbers exceeding their capacity [13–15]. There are at least two reasons for this phenomenon. First, the patients suffering severe injuries need immediate medical interventions, which often necessitate the patient to be transferred to the nearest hospital. Second, decision-making about the distribution of patients is very complicated, and response managers are unable to allocate enough resources for managing the incident [4]. Access to information such as injury severity of casualties, scene evacuation time, and real-time pre-hospital and hospital resources will help decision-makers better understand and manage situation [2, 4, 8, 16].

Regarding to application of the models for casualty distribution following mass casualty incidents, above-mentioned criteria have long been considered important in the management of mass casualty incidents. However, no single model has yet addressed the effects and interaction of these factors on the general decision-making processes. Researchers therefore need to examine and analyze the different factors affecting patient distribution following mass casualty incidents in order to develop and test practical models for this purpose.

The present study is a first step in developing a comprehensive model of casualty distribution following mass casualty incidents. Given the comprehensiveness of the methodology of the study and since it encompasses all the studies conducted on the subject of casualty distribution, it can be used in developing a practical model for the better management of mass casualty incidents.

## Additional file

Additional file 1: PRISMA-P Checklist, Filename: PRISMA-P + checklist. (DOCX 397 kb)
